# Supplementary material for: Understanding patient preferences in anti-VEGF treatment options for age-related macular degeneration
Source: PLoS One. 2022 Aug 11;17(8):e0272301. doi: 10.1371/journal.pone.0272301 (PMC9371344; doi:10.1371/journal.pone.0272301)
Supplement: S1 File — (DOCX) [file pone.0272301.s001.docx]

**SUPPLEMENTARY MATERIAL 1**

**Statistical Analysis**

Analysis of Discrete Choice Experiment (DCE) data is based on random utility model [1]. The model assumes that utility *U_ij_* for person *i* for treatment *j* is the linear addition of a deterministic component *V_ij_* and an unobserved stochastic error component *ε_ij_* and can be modelled as the following:

*U_ij_ = V_ij_ + ε_ij_*

The treatment which provides the highest utility is chosen by the patient. *V_ij_*, the deterministic part of the utility is approximated by the linear combination of the treatment attributes:

*V_ij_ = β’_ij_ X_j_*

Where *X_j_* represents the vector of attributes of treatment *j* and *β’_ij_* is the vector of preference weights. *ε_ij_* is assumed to be independent and identically distributed following an extreme value Type 1 distribution. An alternative specific constant was also included to capture the preferences for the current treatment.

A latent class logit model was used to estimate patient preferences as it allowed for potential for heterogeneous preferences amongst subsets of the participants [2]. The model assumes that the individuals can be sorted into Q classes with different underlying preference weights (*β’_ij_*) across classes but the same within classes. Conditional on being in class, the utility of person *i* for treatment *j* is:

*U_ij_ =* $\beta_{c}$*X_ij_ + ε_ij_*

The model was estimated using maximum likelihood estimation via the LCLOGIT command in NLOGIT software. Different numbers of total classes Q were tried and the final number was selected based on the stability of the estimates (no unusually large standard errors), not having classes with low prevalence (<10%), prediction error estimates such as the Akaike information criterion and the Bayesian information criterion, and the accuracy of predicting the hold-out task. The associations of class membership probabilities with the sociodemographic, clinical factors (years on medication, fear of injections) and quality of life were also investigated.

**References**

1. McFadden, D., *Conditional logit analysis of qualitative choice behavior.* 1973.

2. Greene, W.H. and D.A.J.T.R.P.B.M. Hensher, *A latent class model for discrete choice analysis: contrasts with mixed logit.* 2003. **37**(8): p. 681-698.
